# Antifouling Compounds from Marine Invertebrates

**DOI:** 10.3390/md15090263

**Published:** 2017-08-28

**Authors:** Shu-Hua Qi, Xuan Ma

**Affiliations:** Key Laboratory of Tropical Marine Bio-resources and Ecology, Guangdong Key Laboratory of Marine Materia Medica, RNAM Center for Marine Microbiology, South China Sea Institute of Oceanology, Chinese Academy of Sciences, 164 West Xingang Road, Guangzhou 510301, China; mx20102112310039@126.com

**Keywords:** marine invertebrate, sponge, coral, antifouling compound

## Abstract

In this review, a comprehensive overview about the antifouling compounds from marine invertebrates is described. In total, more than 198 antifouling compounds have been obtained from marine invertebrates, specifically, sponges, gorgonian and soft corals.

## 1. Introduction

Biofouling includes microfouling (mainly by bacteria and diatoms) and macrofouling (by macro-algae and invertebrates) in the marine environment [1]. Biofouling is a thorny issue that brings tremendous losses in both marine technical and economic fields around the world. In past years, paints containing toxic materials like copper, lead, mercury, arsenic, and organotins such as tributyltin (TBT) were commonly used to control biofouling [2,3]. However, with the increasing global appeal for marine ecological protection, most of these toxic antifouling (AF) coatings were banned [4,5]. It is urgent to have environmentally benign, no or low-toxic AF agents. Marine natural small molecules were secondary metabolites of marine organisms, having the characteristics of high efficiency, low/non-toxicity, being easily degradable, and having less influence on the marine ecological environment, which are thought to be important channels for no or low-toxic AF agents.

Marine invertebrates have developed prominent chemical defense systems against biofouling in the course of evolution. Lots of AF compounds have been isolated from marine invertebrates. Several books [6,7] and reviews [2,8,9,10,11,12,13,14] on AF marine natural products, including compounds from marine invertebrates, have been published in the last 30 years. However, these reviews were partially about some representative AF compounds isolated from marine invertebrates over several years. The review contained in this paper covers almost all of the AF compounds from marine invertebrates from the last 30 years. Its aim is to give the readers a brief, yet comprehensive, overview of AF compounds from marine invertebrates and provide models for synthesis of more efficacious no or low-toxic antifoulants.

## 2. Results

Marine invertebrates, specifically, sponges, gorgonian and soft corals, are rich sources of novel and bioactive secondary metabolites. Studies of the natural chemistry of these interesting groups of marine invertebrates began in the late 1950s. They are recognized to mainly produce novel diterpenoids, sesquiterpenoids, prostanoids, alkaloids, and highly functionalized steroids that are largely unknown from terrestrial sources. Most of these compounds showed AF activity.

### 2.1. Terpenoids

#### 2.1.1. Terpenoids from Sponges

Terpenoids, especially isocyanoterpenoids, were the typical AF metabolites of marine sponges.

**Figure 1:**
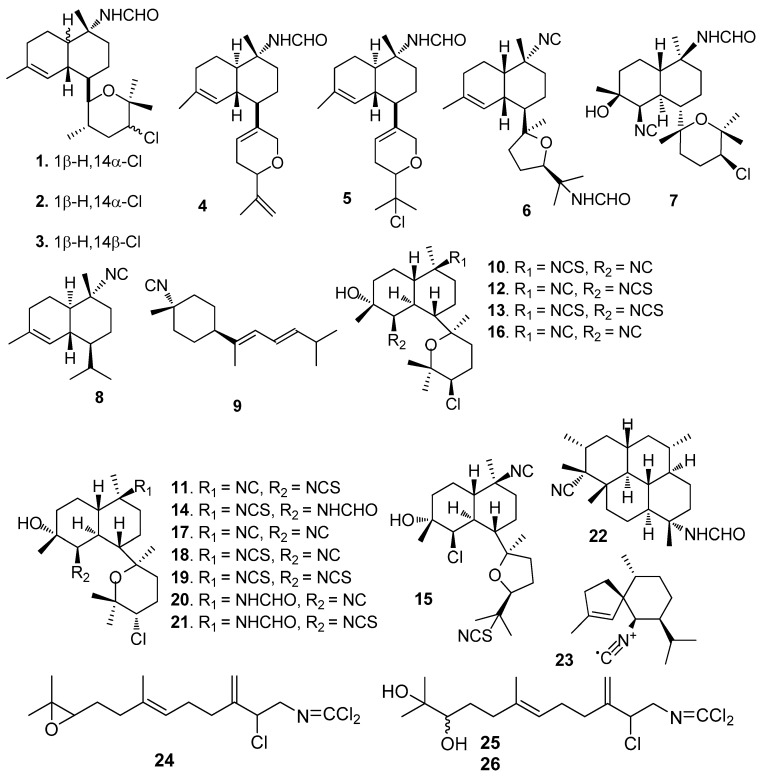
Structures of antifouling (AF) isocyanoterpenoids and analogues from sponges.

AF isocyanoterpenoids and analogues (Figure 1): Kalihinenes X-Z (**l**–**3**) [15] and kalihipyrans A-B (**4**–**5**) [16] were isolated from the marine sponge *Acanthella cavernosa*, showing strong AF activity towards *Balanus amphitrite* (=*Amphibalanus amphitrite*) larvae with EC_50_ values of 0.45–1.3 μg/mL. Isocyanoterpenoids 15-formamidokalihinene (**6**) [16] and 10β-formamidokalihinol A (7) [17] also obtained from *A. cavernosa*, inhibited the *B. amphitrite* larval settlement with EC_50_ < 0.5 μg/mL and low toxicity (LD_50_s > 100 μg/mL). A similar AF activity was found for 10-isocyano-4-cadinene (**8**) and isocyanotheonellin (**9**) that were isolated from nudibranchs of the family Phyllidiidae [18]. Kalihinols M-Q (**10**–**15**) and six analogues (**16**–**21**) were isolated from the Chinese marine sponge *A. cavernosa*, showing significant AF activity against *B. amphitrite* larvae with EC_50_ values of 0.27–1.85 μM [19]. The diterpene isonitrile **22** isolated from *Cymbastela hooperi*, and the sesquiterpene axisonitrile-3 (**23**) isolated from *Acanthella kletra*, were effective in deterring the settlement of the diatom *Nitzschia closterium* [20]. Sesquiterpenes axinyssimides A–C (**24**–**26**) containing a rare dichloromethyleneamino functionality were isolated from a marine sponge *Axinyssa* sp. Among them, **24** inhibited the *B. amphitrite* larval settlement with EC_50_ value of 1.2 μg/mL, and **25** and **26** were more active (EC_50_s < 0.5 μg/mL) [21].

**Figure 2:**
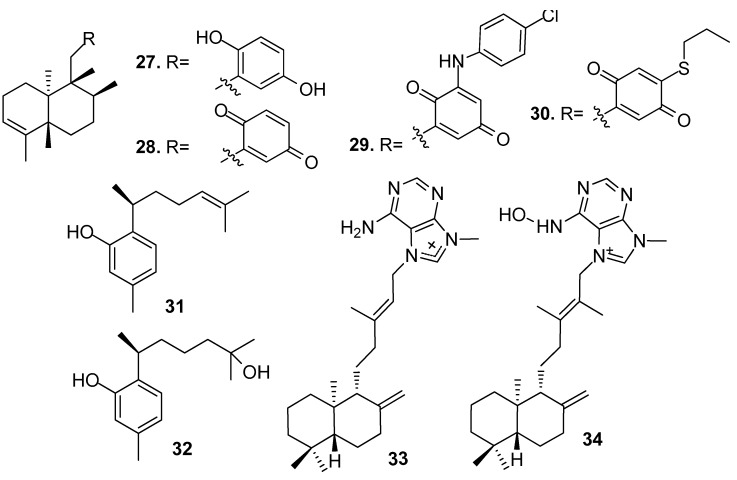
Structures of AF sesquiterpenes and diterpenoids from sponges.

**Figure 3:**
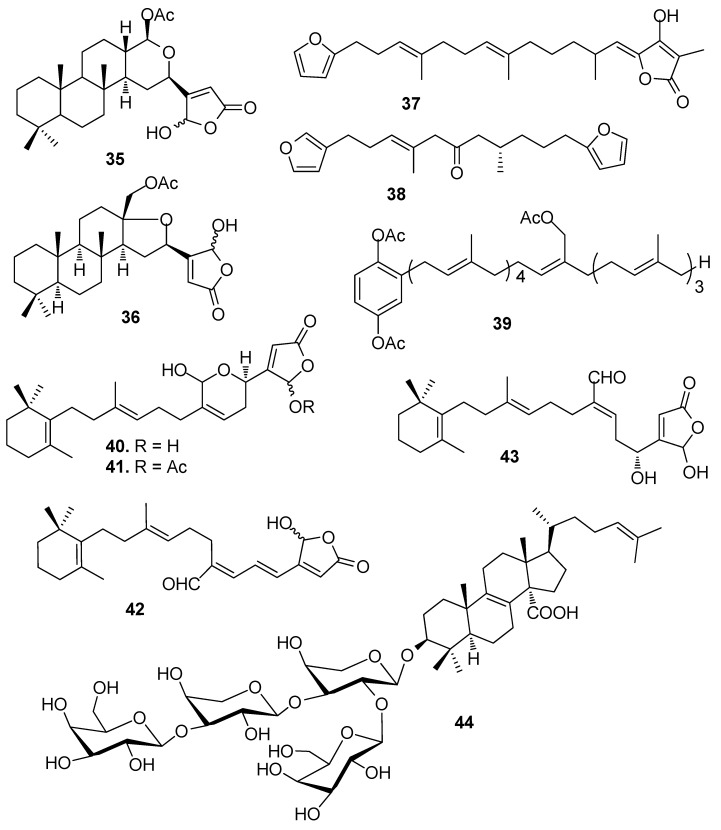
Structures of sesterterpenes and triterpenes from sponges.

Non-isocyanoterpenoids with AF activity from sponges (Figure 2 and Figure 3) included sesquiterpenes, diterpenoids, sesterterpenes, and triterpenes. For examples:

Sesquiterpenes hydroquinone avarol (**27**) and avarone (**28**) obtained from the sponge *Dysidea avara*, and their synthetic analogs 3′-(p-chlorophenyl)avarone (**29**) and 4′-propylthioavarone (**30**) showed strong inhibition against *B. amphitrite* larvae with EC_50_ values of 0.45–3.41 μg/mL [22]. Sesquiterpenes, phenol derivatives (+)-curcuphenol (**31**) and (+)-curcudiol (**32**) from the sponge *Myrmekioderma dendyi* showed antilarval activity against *B. amphitrite* larvae at non-toxic concentrations with EC_50_ values of 2.5 and 2.8 μg/mL, respectively [23].

Diterpenoid alkaloids (−)-agelasine D (**33**) and (−)-ageloxime D (**34**) from an Indonesian sponge *Agelas* sp. showed significant toxicity towards *B. amphitrite* larvae rather than just inhibiting settlement, and the toxicity of **34** was about 10 times than its congener **33**, which indicated the importance of the oxime group for the activity of the diterpene alkaloids. Compound **33** also showed antibacterial activity against the planktonic form of *Staphylococcus epidermidis* (MIC < 0.0877 μM) but did not inhibit its biofilm formation [24].

Sesterterpenes cavernosolide (**35**), lintenolide A (**36**) and 7*E,*12*E,*20*Z*-variabilin (**37**) isolated from the sponge *Semitaspongia bactriana*, showed strong toxicity against the diatom *Nitzschia closterium* and against *Bugula neritina* larvae with EC_50_ values from 1.22 to 7.41 μM [25]. Two analogues of **37**, dihydrofurospongin II (**38**) and hydroquinone-A acetate (**39**) obtained from multiple mediterranean sponge extracts showed significant AF activity against *B. amphitrite* larvae at nontoxic concentrations with EC_50_ values of about 2.5 and 1.0 μg/mL, respectively [26].

Nortriterpenoids manoalide (**40**), *seco*-manoalide (**41**), manoalide 25-acetate (**42**) and (4*E*,6*E*)-dehydromanoalide (**43**) from a sponge *Smenospongia* sp., strongly inhibited the *B. amphitrite* larval settlement at nontoxic concentrations with EC_50_ values of 0.24–2.7 μg/mL [24]. Compound **40** could also inhibit bacterial quorum sensing (QS) at low concentrations [27]. Formoside (**44**), a triterpene glycoside from the sponge *Erylus formosus*, could strongly deter the biofouling of invertebrates and algae [28].

#### 2.1.2. Terpenoids from Corals

The principal terpenoids elaborated by gorgonian and soft corals are sesquiterpenes and diterpenes. The representative structures of diterpenoids by carbon skeleton class from corals included briarane type, cembrane type, eunicellan type, xenicane type, pseudopterosin type, dilophol type, etc. Many of these diterpenoids were reported to have AF activity against marine invertebrate larvae.

**Figure 4:**
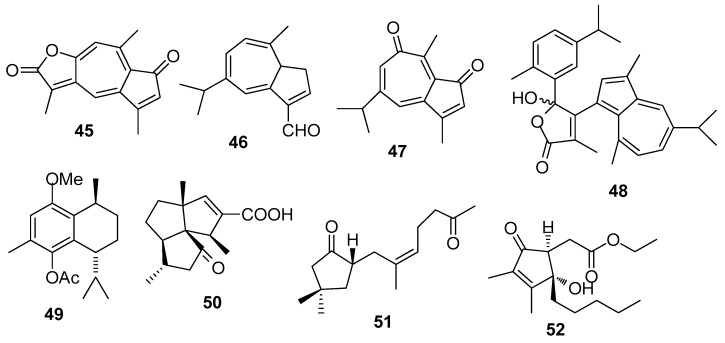
Structures of AF sesquiterpenoids from corals.

AF sesquiterpenoids (Figure 4): Guaiazulene-based terpenoids anthogorgiene G (**45**) and analogus **46**–**48** were isolated from a gorgonian *Anthogorgia* sp., showing inhibition against the larval settlement of *B. amphitrite* larvae with EC_50_ < 7.0 μg/mL [29]. (+)-(7*R*,10*S*)-2-methoxy,5-acetoxy calamenene (**49**) obtained from the octocorals of Indian waters exhibited AF activity against *B. amphitrite* with EC_50_ value of 0.0335 μg/mL [30]. Subergorgic acid (**50**) obtained from the gorgonian *Subergorgia suberosa* showed inhibition against the larval settlement of both *B. amphitrite* and *B. neritina* larvae with EC_50_ values of 1.2 and 3.2 μg/mL, respectively [31]. Sinularones A–B (**51**–**52**) from a soft coral *Sinularia* sp. showed medium AF activity against *B. amphitrite* larvae [32].

**Figure 5:**
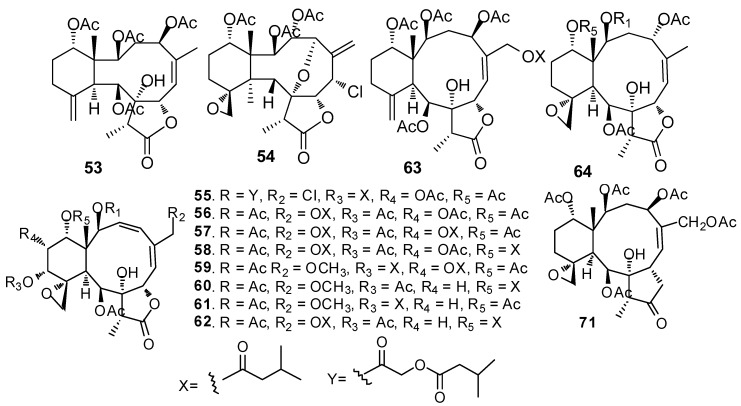

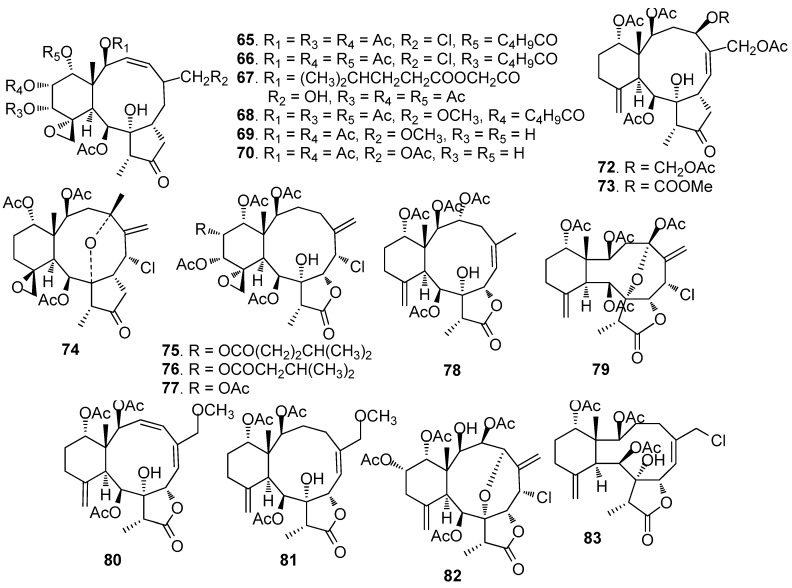
Structures of AF briarane-type diterpenoids from corals.

AF briarane-type diterpenoids (Figure 5): Junceellolide (**53**) and praelolide (**54**) isolated from the gorgonian *Dichotella gemmacea*, showed medium AF activity against the settlement of *B. amphitrite* larvae [33]. Dichotellides H, I, K-P, U (**55**–**63**) and junceellolide C (**64)** were also isolated from *D. gemmacea*, showing potent AF activity at nontoxic concentrations with EC_50_ values of 0.2–7.6 μg/mL [34]. Juncins R-ZI (**65**–**74**), juncin ZII (**75**), gemmacolide B (**76**), gemmacolide A (**77**) and junceellolide D (**78**) were isolated from the gorgonian *Junceella juncea*, showing potent AF activity against *B. amphitrite* larvae at nontoxic concentrations with EC_50_ values from 0.004 to 21.06 μg/mL [35,36]. Briaranes (+)-junceellolide A (**79**), fragilisinins E (**80**), F (**81**) and J (**82**) from *J. fragilis* showed AF activity against *B. amphitrite* larvae with EC_50_ values of 5.6–14.0 µM and low toxicity [37]. Reticulolide (**83**) obtained from the gorgonian *S. mollis* showed strong inhibition against the larval settlement of *B. amphitrite* larvae with EC_50_ value of 0.35 μg/mL [38].

**Figure 6:**
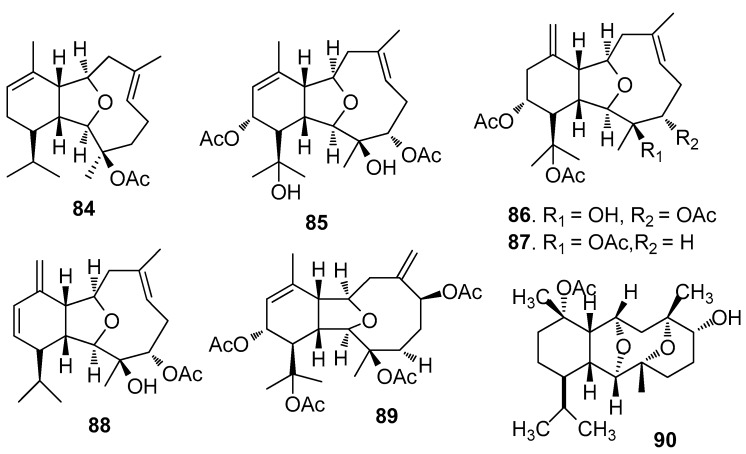
Structures of AF eunicellin-based diterpenoids from corals.

AF eunicellin-based diterpenoids (Figure 6): 14-Deacetoxycalicophirin B (**84**), astrogorgins B-D (**85**–**87**), and analogues **88**–**89** isolated from a gorgonian *Astrogorgia* sp., exhibited AF activity against *B. amphitrite* larvae with EC_50_ values of 0.59–17.8 μg/mL [39]. (−)-6α-Hydroxypolyanthelline A (**90**) from the soft coral *Cladiella krempfi* showed toxicity and AF activity against *B. amphitrite* larvae [40].

**Figure 7:**
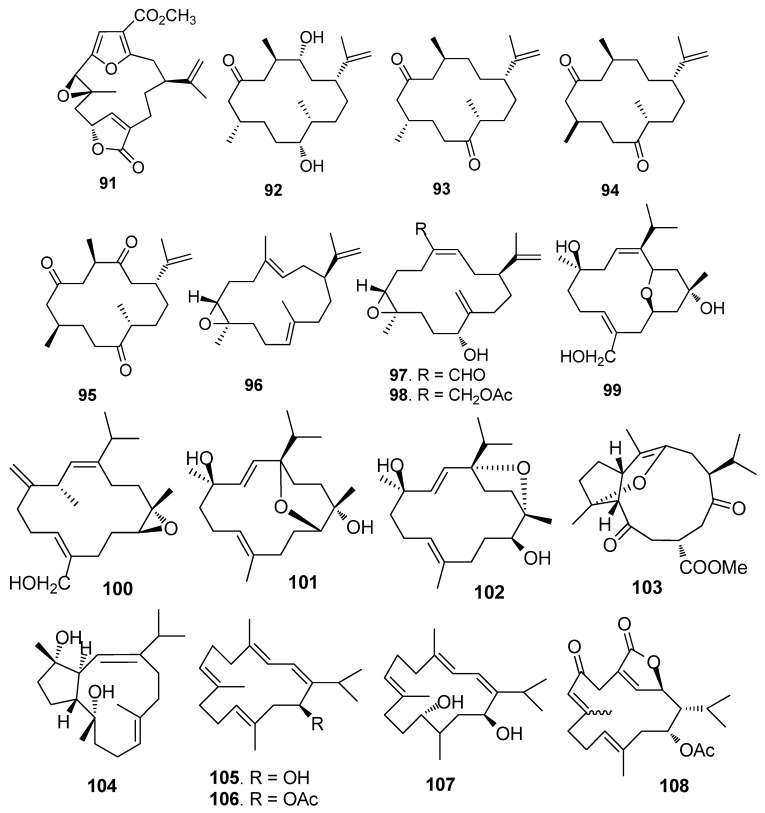
Structures of AF cembrane-type diterpenoids from corals.

AF cembrane-type diterpenoids (Figure 7): Pukalide (**91**) from the gorgonian *Leptogorgia virgulata* showed strong inhibition against the larval settlement of *B. amphitrite* larvae with EC_50_ value of 19 ng/mL [41]. Cembranoid epimers **92**–**95** isolated from the Colombian Caribbean gorgonian *Pseudoplexaura flagellosa*, could inhibit the biofilm maturation of *Pseudomonas aeruginosa*, *Vibrio harveyi*, and *Staphylococcus aureus* without interfering the bacterial growths [42]. Knightine (**96**), 11(*R*)-hydroxy-12(20)-en-knightal (**97**), and 11(*R*)-hydroxy-12(20)-en-knightol acetate (**98**) from the gorgonian *Eunicea knighti*, disrupted QS systems and showed anti-film activity against the bacterial biofilm of *P. aeruginosa*, *V. harveyi*, and *S. aureus* at lower concentrations than kojic acid [43]. Sinulariols J (**99**), P (**100**), Y (**101**) and its analogue **102** from the soft coral *Sinularia rigida* showed potent AF activity against the larval settlement of *B. amphitrite* and *B. neritina* larvae with EC_50_ < 14.03 μg/mL [44,45]. Pavidolides C-D (**103**–**104**) from the soft coral *S. pavida* exhibited inhibition against the larval settlement of *B. amphitrite* larvae with ED_50_ values of 4.32 and 2.12 μg/mL and low cytotoxicity (LD_50_ > 50 μg/mL) [46]. Four cembrene diterpenoids **105**–**108** from the soft coral *Sarcophyton infundibuliforme* showed significant inhibition against the settlement of *B. amphitrite* larvae at nontoxic concentrations [47].

#### 2.1.3. Terpenoids from Other Marine Invertebrates

**Figure 8:**
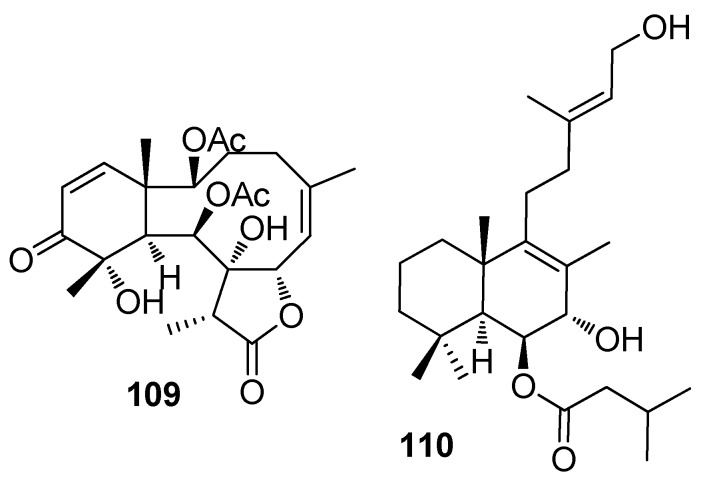
Structures of AF terpenoids from other marine invertebrates.

Briarane-type diterpenoids renillafoulins A (**109**) (Figure 8), B, and C from the sea pen *Renilla reniformis* showed strong inhibition against the barnacle settlement with EC_50_ values ranging 0.02–0.2 μg/mL [48,49]. A labdane diterpene **110** from the pulmonate limpet *Trimusculus reticulatus* could inhibit the settlement of *Phragmatopoma californic*a larvae at 10 μg/mL, and its lethal concentration to the larvae was 100 μg/mL [50].

### 2.2. Steroids and Saponins

#### 2.2.1. Steroids from Sponges

**Figure 9:**
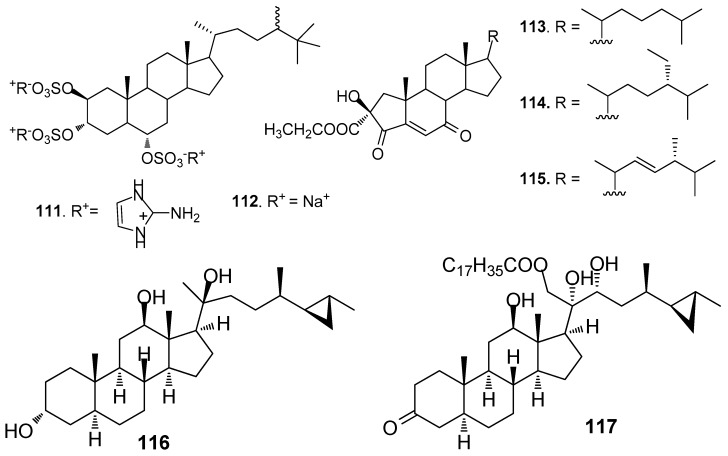
Structures of AF steroids from sponges.

Two steroids tri-2-aminoimidazolium halistanol sulfate (**111**) and halistanol sulfate (**112**) (Figure 9) from a marine sponge *Topsentia* sp, showed AF activity but no toxicity against *B. amphitrite* larvae with EC_50_ values of 4.0 and 2.9 μg/mL, respectively [23]. Three new A-nor steroids, the ethyl esters of 2β-hydroxy-4,7-diketo-A-norcholest-5-en-2-oic acid (**113**), 24S-ethyl-2β-hydroxy-4,7-diketo-A-norcholest-5-en-2-oic acid (**114**), and 2β-hydroxy-4,7-diketo-24*R*-methyl-A-norcholest-5,22(*E*)-dien-2-oic acid (**115**) from the Chinese marine sponge *Acanthella cavernosa* showed medium AF activity against *B. albicostatus* larvae [51]. Cyclopropanated sterols aragusterol I (**116**) and 21-*O*-octadecanoyl-xestokerol A (**117**) isolated from the sponge *Xestospongia testudinaria*, inhibited the growth of *Pseudoalteromonas* and *Polaribacter* bacterial species at similar levels of activity to the positive control tributyltin oxide [52].

#### 2.2.2. Steroids from Coals 

**Figure 10:**
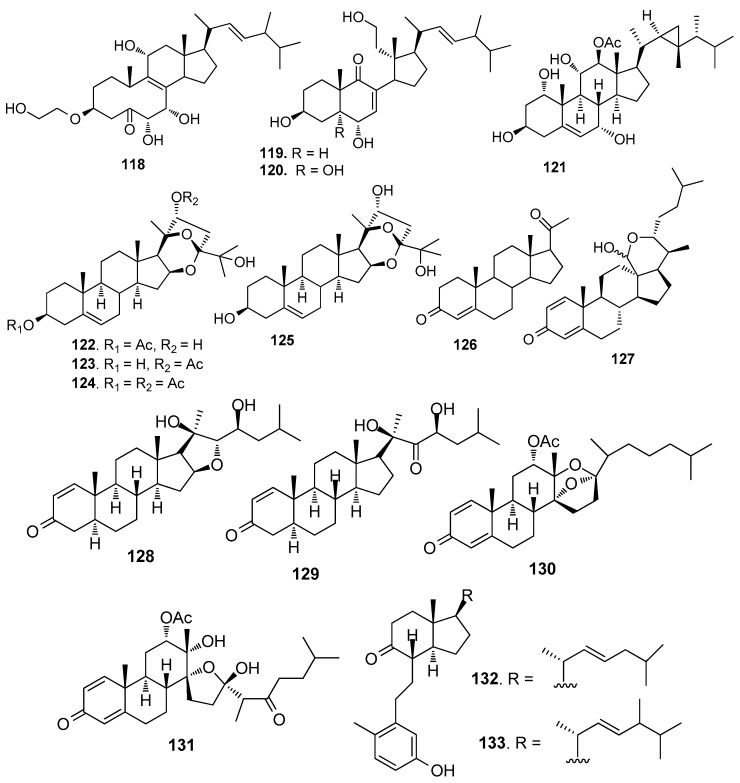
Structures of AF steroids from corals.

Steroids **118** and **119** (Figure 10) from the gorgonian *S. suberosa* inhibited the settlement of *B. neritina* larvae with EC_50_ values of 6.25 and 7.8 μg/mL, respectively, and LD_50_ > 250 μg/mL [53]. Compound **120** was a 5a-hydroxylated analog of **115**, having similar AF activity against *B. neritina* larvae and *B. amphitrite* larvae [51]. 1α,3β,7α,11α,12β)-Gorgost-5-ene-1,3,7,11,12-pentol 12-acetate (**121**) from the gorgonian *Isis minorgrachyblasta* inhibited the settlement of *B. neritina* larvae with EC_50_ value of 4.8 μg/mL and LC_50_ >100 μg/mL [54]. Four 24-ketal steroids (**122**–**125**) from the gorgonian *S. mollis* showed AF activity against *B. amphitrite* larvae at nontoxic concentrations with EC_50_ values of 0.81–7.91 μg/mL [39]. Pregn-4-ene-3,20-dione (**126**) showed medium AF activity against the larval settlement of both *B. amphitrite* and *B. neritina* larvae [31]. A pentacyclic hemiacetal sterol nephthoacetal (**127**) from a soft coral *Nephthea* sp. showed significant AF activity against *B. amphitrite* larvae with EC_50_ value of 2.5 μg/mL and LC_50_ > 25.0 μg/mL [55]. Two cholestane derivatives, pentacyclic steroid 16,22-epoxy-20β,23S-dihydroxycholest-1-ene-3-one (**128)** and 20β, 23S-dihydroxycholest-1-ene-3,22-dione (**129**) from the gorgonian *S. suberosa* showed potent inhibition activity towards the settlement of *B. amphitrite* larvae [56]. Unprecedented D-secosteroids, isogosterones A (**130**) and C (**131**) isolated from a soft coral *Dendronephthya* sp. exhibited AF activity against *B. amphitrite* larvae with EC_50_ value of 2.2 μg/ mL. 9,10-Secosteroids (**132**–**133**) from the gorgonian *Muricella sibogae* showed medium inhibition against the settlement of *B. amphitrite* larvae [57].

### 2.3. Alkaloids

Many types of AF alkaloids, especially brominated alkaloids, have been isolated from marine sponges. 

**Figure 11:**
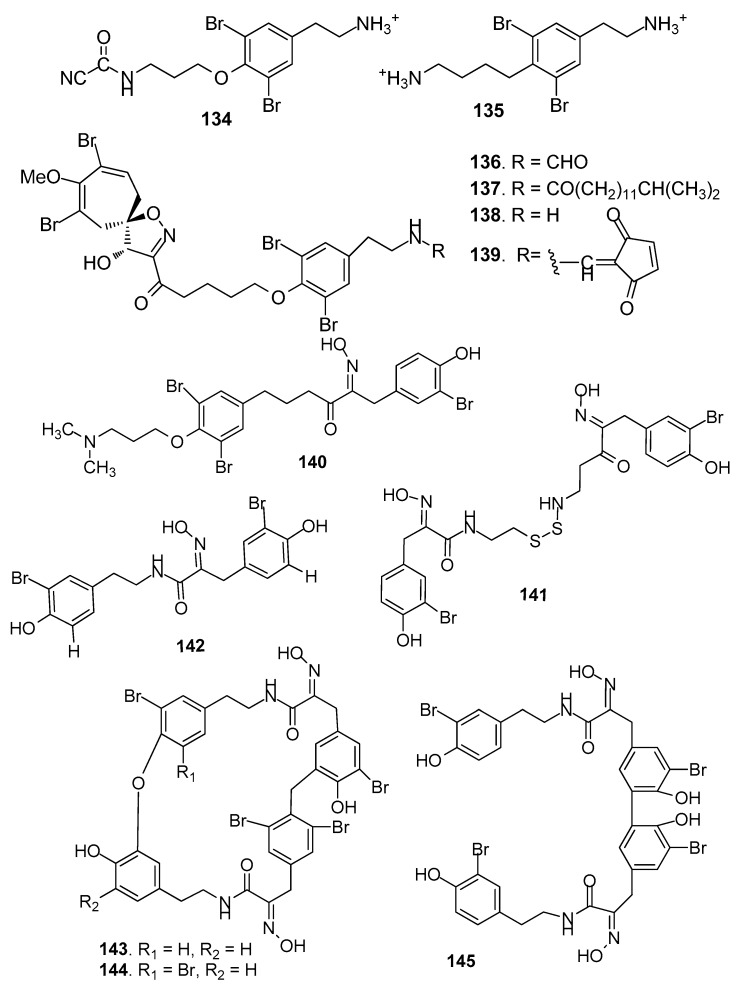
Structures of AF bromotyrosine-derived compounds from sponges.

AF bromotyrosine-derived compounds (Figure 11): Bromotyrosine-derived compounds were specially found in marine sponges of the families Aplysinidae and Pseudoceratinidae, particularly *Pseudoceratina* (=*Psammaplysilla*) *purpurea*. Ceratinamine (**134**) [58], moloka’iamine (**135**) [59], ceratinamides A-B (**136**–**137**) [58], and psammaplysins A (**138**) and E (**139**) [58] were isolated from the sponge *P. purpurea*, showing AF activity against *B. amphitrite* cyprids with EC_50_ values ranging from 0.10 to 8.0 μg/ mL [58]. The AF activities of aplysamine-2 (**140**) from *P. purpurea*, a synthetized analog hemibastadin-1 (**141**), psammaplins A (**142**) from *Aplysinella rhaxand*, and three bastadins-9, -16, -3 (**143**–1**45)** derivatives from *Ianthella basta* were also evaluated. Among them, **140** and **143**–1**45** could significantly inhibit the settlement of *B. amphitrite* larvae at concentrations of 1 or 10 μM without increasing larval mortality, while **141**, **142** and **144** showed inhibition against larval settlement at 10 μM with significant mortality of the cyprids [60].

**Figure 12:**
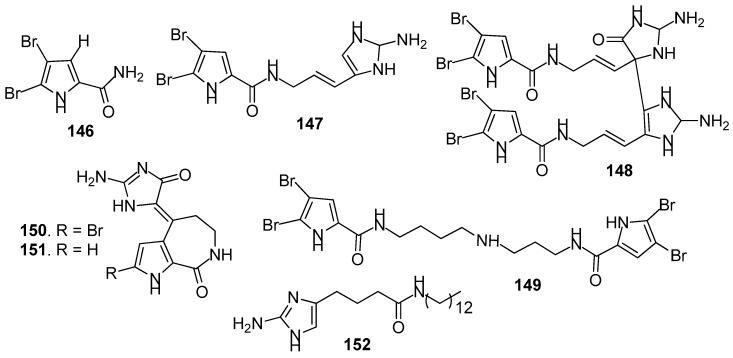
Structures of AF pyrrole-derived compounds from sponges.

AF pyrrole-derived compounds (Figure 12): Bromopyrrole-derived compounds 4,5-dibromopyrrole-2-carbamide (**146**), oroidin (**147**) and mauritiamine (**148**) were isolated from the sponge *Agelas mauritiana*. Compounds **147** and **148** showed medium inhibition against the larval metamorphosis of *B. amphitrite* larvae, while **146** could promote the larval metamorphosis of the ascidian *Ciona savignyi* at 2.5 µg/mL [61]. A spermidine derivative pseudoceratidine (**149**) from *P. purpurea* showed AF activity against *B. amphitrite* larvae [62]. Hymenialdisine (**150**) and debromohymenialdisine (**151**) isolated from a sponge *Axinella* sp. were found to exhibit significant AF activity against the green mussel *Perna viridis*, the bryozoan *B. neritina*, and the green alga *Ulva. prolifera* [63]. A pyrroloimidazole alkaloid **152** isolated from sponge, showed significant inhibition against the bacterial attachment of *Pseudomonas* with IC_50_ value of 0.73 μM [64].

**Figure 13:**
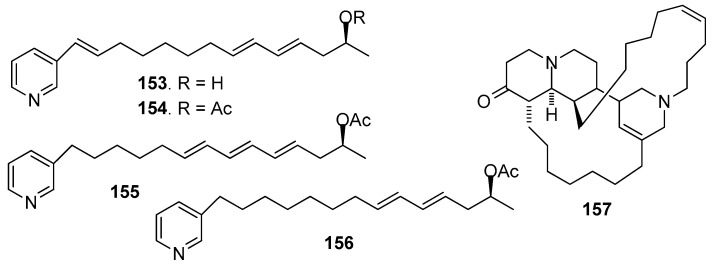
Structures of AF pyridine-derived compounds from sponges.

AF pyridine-derived compounds (Figure 13): Two synthetic compounds haminol-A (**153**) and haminol-B (**154**), and three natural compounds haminol-2 (**155**), haminol-4 (**156**) and saraine-1 (**157**) from *Haliclona fusari* were evaluated for their AF activity, which showed that **153**–**157** significantly inhibited the larval settlement of *B. amphitrite* larvae with EC_50_ values ranging from 0.28 to 3.6 μg/mL [65].

**Figure 14:**
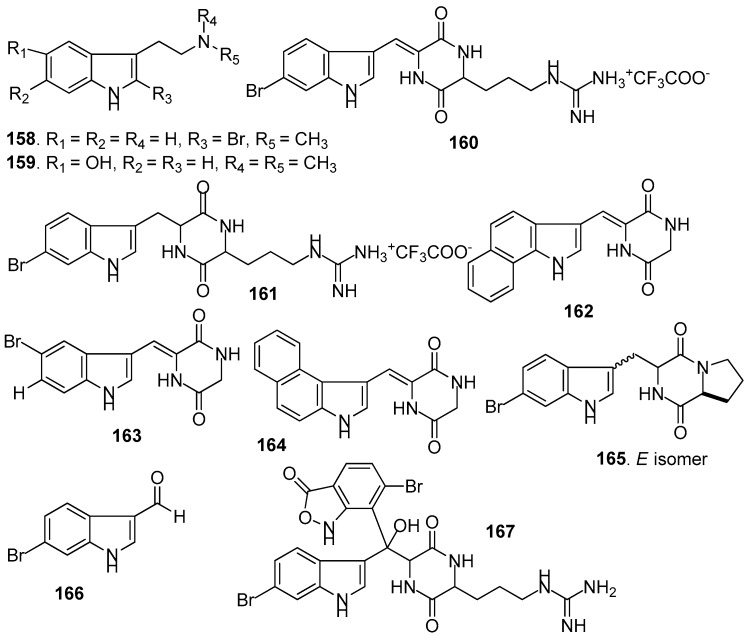
Structures of AF indole alkaloids from sponges.

AF indole alkaloids (Figure 14): Alkaloids 2-bromo-*N*-methyltryptamine **158**–**159** from the gorgonian *Paramuricea clavata* showed significant anti-adhesion activity against one marine bacterial strain with nontoxicity [66]. Barettin (**160**) and 8,9-dihydrobarettin (**161**) from the sponge *Geodia barretti* showed inhibition against the settlement of *B. improvises* larvae with EC_50_ values of 0.9 and 7.9 µM, respectively [67]. In 2006, 14 analogs of **161** were synthesized. Among them, benzo[*g*]dipodazine (**162**) and other four dipodazine analogs (**163**–**166**) with a dipodazine group significantly inhibited the settlement of *B. improvisus* larvae with EC_50_ values of 0.034, 5.8, 1.5, 2.4 and 6.7 μM [68], respectively. Bromobenzisoxazolone barettin (**167**) from the sponge *G. barrette* inhibited the settlement of *B. improvisus* larvae with EC_50_ value of 15 nM [69].

**Figure 15:**
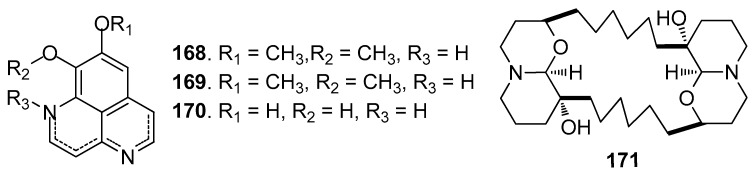
Structures of other kinds of AF alkaloids from sponges.

Other AF alkaloids (Figure 15): Aaptamine (**168**), isoaaptamine (**169**), and demethylated aaptamine (**170**) isolated from the sponge *Aaptos aaptos* showed AF activity against zebra mussel attachment [70]. A fraction of the acetone extract of the sponge *Haliclona exigua* was rich in bis-1-oxaquinolizidine alkaloid (**171**), exhibiting significant AF activity against the growths of seven fouling bacterial strains and against the settlement of *B.* *amphitrite* larvae [71].

### 2.4. Other Kinds of Compounds

Besides the above characteristic terpenoids, alkaloids and steroids, there were many other kinds of AF compounds isolated from marine invertebrates, such as polyacetylenes, butenolides, phenol derivatives, and peptides.

**Figure 16:**
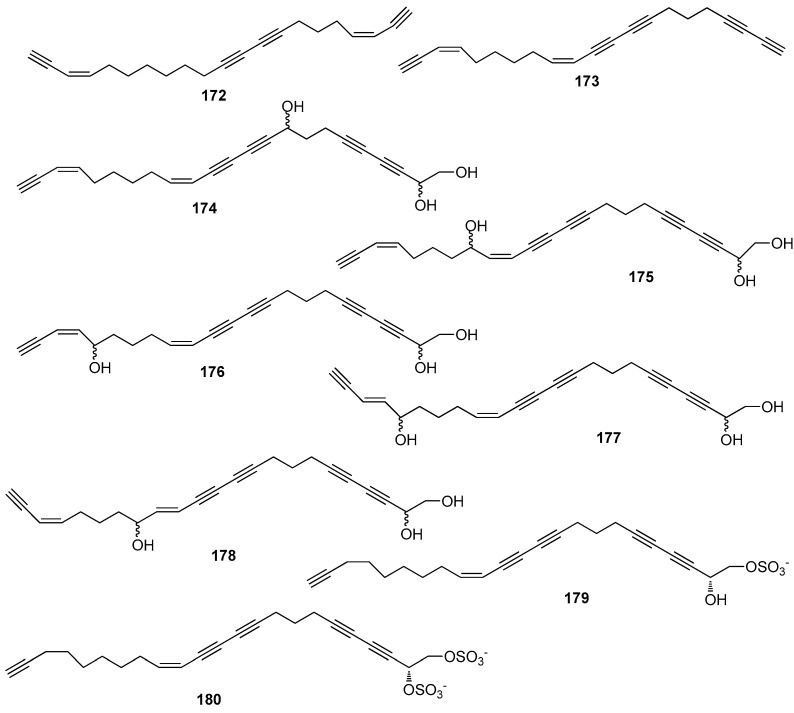
Structures of AF polyacetylene derivatives from sponges.

AF polyacetylene derivatives (Figure 16): Callytetrayne (**172**), callypentayne (**173**), callytriols A-E (**174**–**178**) and callyspongins A-B (**179**–**180**) from the sponge *Callyspongia truncate* showed potent metamorphosis-inducing activity towards the ascidian *Halocynthia roretzi* larvae with ED_100_ values of 0.13–1.3 µg/mL, and **174**–**180** also showed AF activity against *B. amphitrite* larvae with ED_50_ values of 0.24–4.5 µg/mL [72].

**Figure 17:**
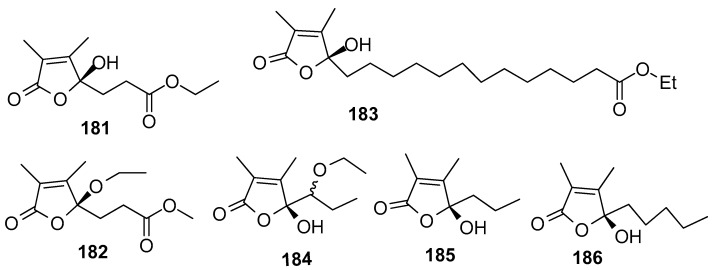
Structures of AF polyacetylene derivatives from sponges.

AF butenolides (Figure 17): Sinularones G-I (**181**–**183**) from a soft coral *Sinularia* sp. showed moderate AF activity against the barnacle *B. amphitrite* [32]. Butenolide (5*R*)-5-(1-ethoxypropyl)-5-hydroxy-3,4-dimethylfuran-2(5*H*)-one (**184**) as a pair of inseparable epimers, along with (*S*)-5-hydroxy-3,4-dimethyl-5-propylfuran-2(5*H*)-one (**185**) and (*S*)-5-hydroxy-3,4-dimethyl-5-pentylfuran-2(*5H*)-one (**186**) were obtained from the gorgonian *S. suberosa*. Compounds **184**–**186** exhibited moderate AF activity against the settlement of *B. amphitrite* larvae [73]. The structure–activity relationship indicated that α,β-unsaturated 2,3-dimethyl-γ-lactone was a functional unit for the antilarval activity.

**Figure 18:**
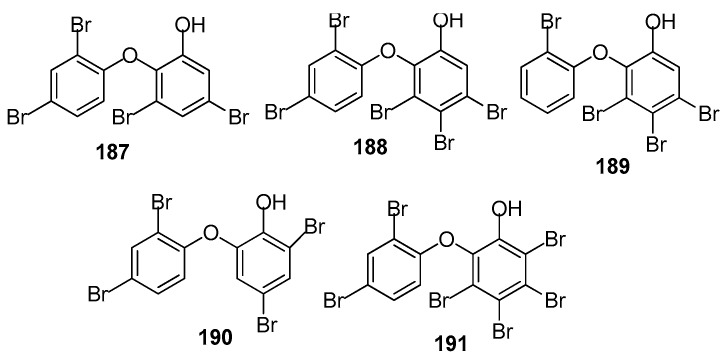
Structures of AF brominated phenol derivatives from sponges.

AF brominated phenol derivatives (Figure 18): Brominated diphenyl ethers are the characteristic secondary metabolites of the genus *Dyside*. It was believed that this type of compound was biosynthesised by the symbiotic cyanobacteria of the sponge. Five polybrominated diphenyl ethers including **187** from a sponge *Callyspongia* sp., **188** from *Dysidea granulosa*, and **189**–**191** from *D. herbacea* were investigated against several taxa of prominent fouling organisms including marine bacteria, the diatom *A. coffeaeformis*, the barnacle *B. amphitrite* and the mussel *Mytilus edulis*. All of these compounds exhibited significant antibacterial and AF activity. Compound **187** was the strongest in all the bioassays with non-toxicity. It inhibited the growth of all of the tested bacterial strains with MIC ¼ 0.02–1.52 µM, and inhibited the larval settlement of *A. coffeaeformis*, *B. amphitrite* and *M. edulis* larvae with EC_50_ values of 0.24, 0.66 and 1.26 µM, respectively [74].

**Figure 19:**
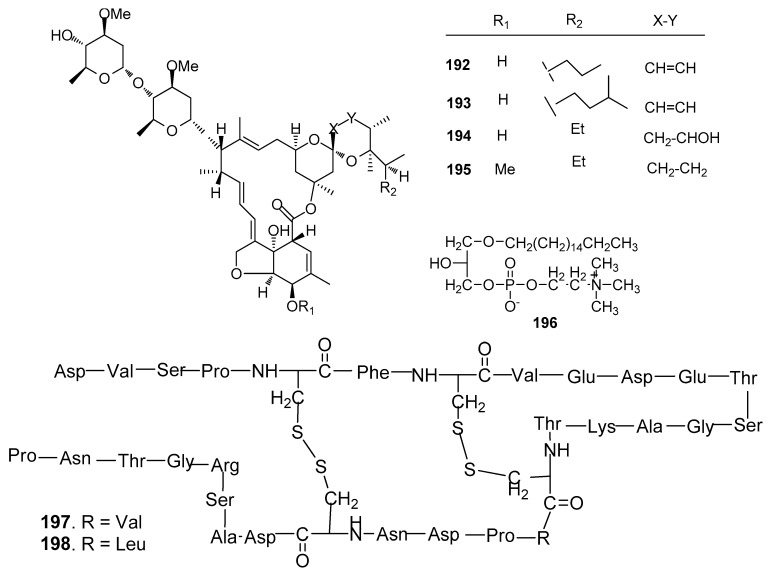
Structures of other kinds of AF compounds from sponges and corals.

Other AF compounds (Figure 19): Four avermectin derivatives, avermectins B_1c_ and B_1e_ (**192** and **193**), avermectin B_2a_ (**194**) and ivermectin A_1a_ (**195**) from the gorgonian *Anthogorgia caerulea* exhibited potent antilarval activity towards *B. amphitrite* larvae with low-toxicity [75]. 1-*O*-palmityl-*sn*-glycero-3-phosphocholine (**196**) from the sponge *Crella incrustans* showed strong inhibition against the settlement of *B. amphitrite* larvae [76]. Two novel disulfide-containing peptides, barrettides A (**197**) and B (**198**) from the sponge *Geodia barrette* showed significant antilarval activity against the settlement of *B. improvises* larvae at concentrations of 0.6 and 6 μM, respectively [77].

## 3. Conclusions

Totally, over 198 AF compounds have been obtained from marine invertebrates, especially, sponges, gorgonian and soft corals. These compounds covered isocyanoterpenoids, sesquiterpenes, diterpenes, sesterterpenes, triterpenoids, alkaloids (including bromotyrosine-derived, pyrrole-derived, pyridine-derived and indole-derived compounds), steroids, polyacetylenes, butenolides, peptides, and phenol derivatives, which played important chemical defense roles in the marine invertebrates. In here, the AF activities of 198 compounds towards microfouling and macrofouling were summarized in Table 1. It is thought that AF compounds have medium to high bioactivity with a threshold of EC_50_ < 15 μg/mL, and AF compounds having high LC_50_/EC_50_ ratios (>15) are potentially good candidate antifoulants [14]. From Table 1, we can see that some of these compounds are potent antifoulants with low/non-toxicity, such as some of the isocyanoterpenoids, briarane-type diterpenoids, cembrane-type diterpenoids, and indole alkaloids. However, little was known about their mode of actions and AF activities in fields, because of the serious problems of the supplies from these marine invertebrates, which restricted the development of these potent AF compounds in antifouling paints. Although some studies about the total synthesis of several isocyanoterpenoids, briarane-type diterpenoids, and cembrane-type diterpenoids have been done, too many steps of these synthetic routes with low yields limited their applications. To overcome the problems, more studies about the organic syntheses of these potent AF compounds as models are needed. In addition, scientists have paid more attention to AF compounds from marine microorganisms, especially sponge-derived and gorgonian-derived microorganisms in recent years.

## Figures and Tables

**Table 1:** AF activities of **1**–**198** towards microfouling (mainly by bacteria and diatoms) and macrofouling (mainly by *B. amphitrite*, *B. albicostatus, B. improvises, B. neritina*, *M. edulis*, *P. viridis* or *H. roretzi*).

Compounds	AF Activity
**1**–**5**	against *B. amphitrite* larvae, EC_50_ = 0.49, 0.45, 1.1, 1.3, 0.85 μg/mL
**6**–**9**	against *B. amphitrite* larvae, EC_50_ < 0.5 μg/mL
**10**–**21**	against *B.* *amphitrite* larvae, EC_50_ = 1.43, 0.72, 1.48, 1.16, 0.53, 0.74, 1.85, 0.92, 0.69, 0.27, 1.37, 0.41 μM
**22**–**23**	effective in deterring the settlement of the diatom *N.* *closterium*
**24**–**26**	against *B. amphitrite* larvae, EC_50_ = 1.2, <0.5, <0.5 μg/mL
**27**–**30**	against *B. amphitrite* larvae, EC_50_ = 0.65, 3.41, 0.65, 0.45 μg/mL
**31**–**32**	against *B. amphitrite* larvae, EC_50_ = 2.5, 2.8 μg/mL
**33**–**34**	significant antilarval activity and toxicity towards *B. amphitrite* larvae
**35**–**37**	toxicity against the diatom *N*. *closterium* with EC_50_ = 5.24, 6.72, 3.52 μM, and against *B. neritina* larvae with EC_50_ = 1.59, 7.41, 1.22 μM
**38**–**39**	against *B. amphitrite* larvae, EC_50_ = 2.5, 1.0 μg/mL
**40**–**43**	against *B. amphitrite* larvae, EC_50_ = 0.24, 0.80, 0.53, 2.7 μg/mL
**44**	strongly deter fouling by invertebrates and algae
**45**–**48**	against *B. amphitrite* larvae, EC_50_ < 7.0 μg/mL
**49**	against *B. amphitrite* larvae, EC_50_ = 0.0335 μg/mL
**50**	against *B. amphitrite* larvae, EC_50_ = 1.2 μg/mL; against *B. neritina* larvae, EC_50_ = 3.2 μg/mL
**51**–**52**	against *B. amphitrite* larvae, EC_50_ = 13.86, 23.50 μg/mL
**53**–**54**	against *B. amphitrite* larvae, EC_50_ =14.5, 16.7 µM
**55**–**64**	against *B. amphitrite* larvae, EC_50_ = 4.1, 1.82, 6.3, 7.6, 4.6, 1.2, 5.6, 0.79, 2.0, 0.2 μg/mL
**65**–**78**	against *B. amphitrite* larvae, EC_50_ = 0.004, 0.34, 2.65, 1.61, 3.77, 21.06, 0.004, 0.14, 1.47, 0.51, 0.004, 0.005, 2.82, 0.447 μg/mL
**79**–**82**	against *B. amphitrite* larvae, EC_50_ = 5.6, 14.0, 12.6, 11.9 µM, LC_50_/EC_50_ > 33.3, > 13, > 14.5, > 11.5, respectively
**83**	against *B. amphitrite* larvae, EC_50_ = 0.35 μg/mL
**84**–**89**	against *B. amphitrite* larvae, EC_50_ = 0.59, 5.77, 5.14, 8.23, 10.7, 17.8 μg/mL
**90**	against *B. amphitrite* larvae, EC_50_ = 9.02 μg/mL, LC_50_ = 36 μg/mL
**91**	against *B. amphitrite* larvae, EC_50_ = 19 ng/mL
**92**–**95**	exhibited inhibition of biofilm maturation of *P.* *aeruginosa*, *V.* *harveyi*, and *S.* *aureus*
**96**–**98**	showed bacterial biofilm inhibition at lower concentrations
**99**–**100**	against *B. amphitrite* larvae, EC_50_ = 5.65, 14.03 μg/mL
**101**–**102**	against *B. amphitrite* larvae, EC_50_ = 4.86, 4.57 μg/mL; against *B. neritina* larvae, EC_50_ = 12.34, 13.48 μg/mL
**103**–**104**	against *B. amphitrite* larvae, ED_50_ = 4.32, 2.12 μg/mL, LD_50_ > 50 μg/mL
**105**–**108**	against *B. amphitrite* larvae, EC_50_ = 2.25, 1.75, 8.13, 7.50 μg/mL
**109**	against *B. amphitrite* larvae, EC_50_ values ranging 0.02–0.2 μg/mL for **109** and renillafoulins B–C
**110**	inhibited the settlement of the tube worm *P. californic*a at 10 μg/mL
**111**–**112**	against *B. amphitrite* larvae, EC_50_ = 4.0, 2.9 μg/mL
**113**–**115**	against *B. albicostatus* larvae, EC_50_ = 8.2, 23.5, 31.6 μg/mL
**116**–**117**	inhibited the growth of *Pseudoalteromonas* and *Polaribacter* bacterial species
**118**–**120**	against *B. neritina* with EC_50_ = 6.25, 7.8 μg/mL, LD_50_ > 250 μg/mL
**121**	against *B. neritina* larvae, EC_50_ = 4.8 μg/mL, LC_50_ > 100 μg/mL
**122**–**125**	against *B. amphitrite* larvae, EC_50_ = 2.5, 7.91, 7.31, 0.81 μg/mL
**126**	against *B. amphitrite* larvae, EC_50_ = 16.7 μg/mL; against *B. neritina* larvae, EC_50_ = 13.0 μg/mL
**127**	against *B. amphitrite* larvae, EC_50_ = 2.5 μg/mL, LC_50_ > 25.0 μg/mL
**128**–**129**	against *B. amphitrite* larvae, EC_50_ = 5.3, 14.5 μg/mL
**130**–**131**	against *B. amphitrite* larvae, EC_50_ = 2.2 μg/mL
**132**–**133**	against *B. amphitrite* larvae, EC_50_ = from 10.0 to 50.0 μg/mL
**134**–**135**	against *B. amphitrite* larvae, EC_50_ = 5.0, 4.3 µg/mL
**136**–**139**	against *B. amphitrite* larvae, ED_50_ = from 0.10 to 8.0 μg/ mL
**140**–**145**	inhibited *B. amphitrite* larval settlement at 1 or 10 μM
**146**	promoted larval metamorphosis of the ascidian *C. savignyi* at a concentration of 2.5 µg/mL
**147**–**148**	inhibited the larval metamorphosis of *B. amphitrite* larvae, ED_50_ = 19, 15 µg/mL
**149**	against *B. amphitrite* larvae, EC_50_ = 8.0 µg/mL
**150**–**151**	against the green mussel *P.* *viridis* (EC_50_ = 31.77, 138.18 μg/mL), the bryozoan *B. neritina* (EC_50_ = 3.43, 8.17 μg/mL) and the green alga *U. prolifera* (EC_50_ = 8.31, 0.67 μg/mL)
**152**	inhibited bacterial attachment towards *Pseudomonas* with an IC_50_ = 0.73 μM
**153**–**157**	against *B. amphitrite* larvae, EC_50_ = 2.22, 3.6, 0.28, 2.81, 0.53, μg/mL
**158**–**159**	anti-adhesion activity against one marine bacterial strain
**160**–**161**	against *B. improvises* cyprids, EC_50_ = 0.9, 7.9 µM
**162**–**166**	against *B. improvises* cyprids, EC_50_ = 0.034, 5.8, 1.5, 2.4, 6.7 μM
**167**	against *B. improvises* cyprids, EC_50_ =15 nM
**168**–**170**	against zebra mussel attachment with EC_50_ = 24.2, 11.6, 18.6 μM
**171**	against cyprids of *B. amphitrite* (EC_50_ = 6.6 μg/mL, LC_50_ = 18 μg/mL) and seven strains of fouling bacteria
**172**–**180**	against *B. amphitrite* larvae with ED_50_ = 0.24–4.5 µg/mL for **174**–**180**; and metamorphosis-inducing activity in the ascidian *H. roretzi* larvae with ED_100_ = 0.13–1.3 µg/mL for **172**–**180**.
**181**–**183**	EC_50_ = 18.65, 21.39, 12.58 μg/mL
**184**–**186**	against *B. amphitrite* larvae, EC_50_ = 13.5, 16.3, 12.8 μg/mL
**187**–**191**	significant antibacterial and antifouling activity towards marine bacteria, *A.* *coffeaeformis*, *B. amphitrite* and *M. edulis*
**192**–**195**	against *B. amphitrite* larvae, ED_50_ = 15.81, 6.25, 4.81, 7.78 μg/mL, LD_50_ > 200 µg/mL
**196**	strong inhibition against the settlement of *B. amphitrite* larvae
**197**–**198**	**197** inhibited the settlement of *B. improvises* larvae at both 0.6 and 6 μM, whereas **198** only at 6 μM
